# The Memory of the Heart

**DOI:** 10.3390/jcdd5040055

**Published:** 2018-11-11

**Authors:** Marco Cirillo

**Affiliations:** Heart Failure Surgery Unit, Cardiac Surgery Unit, Cardiovascular Department, Poliambulanza Foundation Hospital, Via Leonida Bissolati 57, 25125 Brescia, Italy; marco.cirillo@poliambulanza.it; Tel.: +39-030-351-8088

**Keywords:** heart embryology, myocardial structure, interlaced myocardial fibers, cardiomyopathy, left ventricular reconstruction, heart failure, valve disease, mitral regurgitation, guidelines, cardiac resynchronization

## Abstract

The embryological development of the heart is one of the most fascinating phenomena in nature and so is its final structure and function. The various ontogenetic passages form the evolutive basis of the final configuration of the heart. Each key step can be recognized in the final features, as the heart maintains a kind of “memory” of these passages. We can identify the major lines of development of the heart and trace these lines up to the mature organ. The aim of this review is to identify these key parameters of cardiac structure and function as essential elements of the heart’s proper functioning and bases for its treatment. We aim to track key steps of heart development to identify what it “remembers” and maintains in its final form as positively selected. A new vision based on the whole acquired knowledge must guide an in-depth scientific approach in future papers and guidelines on the topic and a complete, farsighted therapeutic conduct able to ensure the physiological correction of cardiac pathologies. The application of this modern, functional vision of the heart could improve the clinical treatment of heart disease, filling the gaps still present.

## 1. Introduction

**Figure 1:**
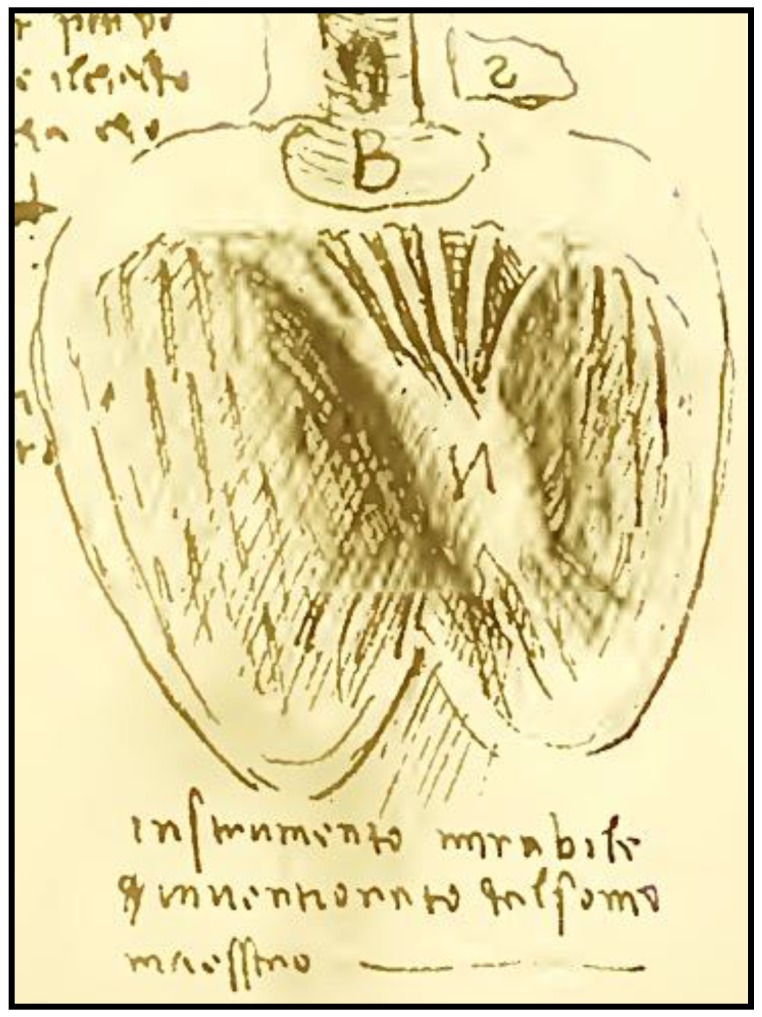
“*Instrumento mirabile invenzionato dal sommo maestro*”, Leonardo da Vinci, Dell’Anatomia, 1489, Fogli B, readable mirror image of Figure 64, foglio 12, recto; public domain electronic copy at: https://archive.org/stream/imanoscrittidile00leon#page/2.

The embryological development of the heart is one of the most fascinating phenomena in nature and so is its final structure and function (Figure 1). The heart undergoes profound changes during its development that mutate it from a contractile tubular form to the complex system of atria and ventricles synchronized with each other in the mature organ. At histological level, we move from a single linear layer of contractile myocells to a 3D system of intertwined fibers with different and progressive starting angles.

Following the theory that ontogenesis summarizes phylogeny, various constructs have been expressed to recognize within the embryological development the bases (and phases) of the final structural and functional aspects, even after the Torrent–Guasp theory that identifies a continuous muscle band that constitutes the whole heart [1]. The demonstration of being able to dissect the heart recognizing this continuous muscular band and carrying it out following its layers made it easy to think of it as the result of the embryological process of cardiac looping. It has not yet been possible to demonstrate a true correlation between the looping process and the final structure of the heart, but it has not even been possible to disprove it [2].

Nevertheless, the various ontogenetic passages form the logical basis of the final structure and function of the heart. Each step can be traced up to its final characteristics, so that we can deduce that the heart maintains a certain “memory” of these passages. We cannot make a close parallelism between each embryological phase and its final result, just as we cannot find all the evolutionary steps in embryonic development, but only those positively selected. However, we can identify the great lines of development of the heart and find these lines (structural and functional) in the final organ.

The aim of this review is to identify and trace these key parameters of cardiac structure and function in order to better understand the essential bases of the heart’s proper functioning. We aim to follow key steps of heart development to identify what the heart “remembers”, what it maintains in its final form as positively selected. This to identify which fundamental parameters we must try to restore in case of heart disease, given that the mature heart preserves just the positively selected elements of its development.

## 2. The Memory, the Heart and the Perception We Have

The memory is what remains forever, what stays during our life (individual memory) and is preserved in the next generations (species memory). The memory is made by experiences and assures the evolution, both of the individual and of the species. In multicellular organisms, all the processes that lead to the development of the embryo are set down in the genetic memory and gene regulation drives cellular differentiation and morphogenesis leading to the creation of different cell types that possess different gene expression profiles from the same genome sequence. This explains how evolution actually works at a molecular level (evolutionary developmental biology) and then brings the functional effects of the molecular structure on a larger scale. In the same way, the scientific approach to any subject requires a revision of what is already known and every potential future improvement starts from it. The “scientific memory” remains of fundamental importance in every new finding.

The evolutionary pathway to mature four-chambered heart is very long and complex and it lasted for the past 600 millions of years [3]. An ontogenetic summary of this long way can be gleaned in the embryologic process of the development of mammalian hearts, that resumes all the phylogenetic steps from the straight tubular vessel of basal chordates to the final pulsating four-chambered heart of mammals [4].

Any single step brings an improvement in efficiency, power, functional anatomy and physiology of the myocardium, driving future developments and assuring blood supply to the increasing metabolic need of more complex living beings. If we go along that path we will see all the basic features of the complex mature heart and recognize the anatomical and functional steps it went through, selecting the most adapted to its function.

Over the centuries the macroscopic anatomical aspect of the heart has been clarified and its function has been correctly framed, but a sort of erroneous vision has lasted up to the present day: that relating to its three-dimensional, integrated, multidimensional structure. Although known since the time of Erysistratus and demonstrated by Harvey (1468), the real three-dimensional structure of the heart muscle was not taken into consideration, nor used in clinical practice if not recently. One of the main reasons for that was the technical limitation of not having an instrumental examination that could show it. Just with the advent of magnetic resonance and 2D echo speckle-tracking and strain [5], we have been able to re-evaluate this fundamental knowledge of cardiac function. Thanks to the more widespread use of these two techniques, an increasing number of literature dealt with the study of cardiac layers structure and function in normal and diseased heart. This has allowed for a new comprehension of several pathological states, suggests a new explanation of systolic and diastolic function/dysfunction and clarified some negative medical and surgical outcomes otherwise inexplicable. As the 3D structure of the myocardium, other structural and functional parameters of the heart also derive from its complex embryological development and all contribute to its normal function: blood flow, conduction system for sequential contraction, valves, chambers’ shape, volumes, synchronism, global and cellular metabolism etc. All these features are histologically intertwined in the myocardial syncytium and structurally related to the whole heart, so that only the sum of all of them can generate a normal cardiac function. The overview of all these parameters is still missing in clinical practice, as several lines of research and study are still struggling to integrate with each other. Retracing the phases of cardiac development by fixing the fundamental features can help us to have a new vision of the heart when analyzing, reporting, treating and operating cardiac pathologies.

## 3. Embryological Development, First Stages

A primitive tubular contracting vessel encounters a looping process on its long axis which approximates opposite regions (inflow and outflow) and creates hollow ventricles from the central portion of original vessel. Lateralization of the heart loop is driven by rotation around its dorsal mesocardium and this has two consequences: lateral displacement of its bending portion and torsion of the cardiac bend into a helical structure. The normal loop presents as a D-loop (towards the right side of the embryo) with left-handed helical winding (left-handed sense of twisting) [6].

### 3.1. The Tubular Embryonic Heart

At the beginning the heart is formed by an inner endothelial tube without valves, a middle layer of extracellular matrix (cardiac jelly), and an outer myocardial tube. This tubular heart starts beating around the 21st day and pushing the blood cells in a laminar, unidirectional way, despite the absence of valves. The two predominant theories about the kind of propulsion of this primitive circulation are that the tubular heart could be a peristaltic or an impedance (Liebau) pump [7]. The embryonic zebrafish heart tubes seem to possess only a single center of active myocardial contractions at its inflow. This part was identified as a region of fast contraction and propagation of the contractile waves (“kickstart” center) [8], whereas the portions downstream to the inflow were identified as regions of decreasing contraction and wave propagation speed. These data suggest that active contraction at the beginning of the tube initiates a wave which then propagates more passively through the rest of the tube [9]. However there is evidence in tubular hearts of higher vertebrate embryos that each segment (inflow, ventricular loop, outflow) is capable of active myocardial contraction, so the mechanical waves traveling along the wall result from active, sequential contractions of the embryonic cardiac segments. We know from in vitro experiments that if an explanted heart tube from a chick embryo is dissected into multiple pieces, each of these physically isolated pieces will undergo active contractions in a segment-specific frequency, which is high in segments derived from proximal (inflow) and low in pieces derived from distal (outflow) heart portion [10].

### 3.2. The Cardiac Jelly

Cardiac jelly is a gelatinous a-cellular material, a relatively homogenous network of collagen fibrils and fine filaments. The structural part of the jelly is ensured by the elastin and collagen scaffold, whereas its gel like appearance is controlled by glycosaminoglycan, a protein involved in the degree of hydration of the jelly. The cardiac jelly is populated by several types of proteins that participate in paracrine cell-to-cell communication, and proteins that promote cell migration and tissue remodeling. There is evidence that the cardiac jelly is a resilient component of the embryonic heart wall, which becomes deformed during systole and springs back to its original shape during diastolic relaxation and, thereby, might suck the blood into the inflow segment of the heart tube [11]. The cardiac jelly shows an uneven spatial distribution, which forces the open lumen of the heart tube to acquire an elliptic cross-section [12,13]. Mathematical analyses have shown that tubes of elliptic cross-section have a higher mechanical efficiency of peristaltic pumping compared with tubes of circular cross-section [14,15].

The traveling contraction waves of the tubular embryonic chick heart thus are accompanied by corresponding waves of lumen occlusion during the initial phase of its pumping activity when all embryonic cardiac segments downstream to the primitive atrium have a thick layer of cardiac jelly. The peristaltic-like pattern of lumen occlusion is present until developmental stages when the embryonic ventricles start formation of a trabeculated muscle layer [16]. The formation of myocardial trabeculations is accompanied by a remarkable thinning of the cardiac jelly in the apical portions of the embryonic ventricles, so that their lumen now is no longer occluded at end-systole (creation of a ventricular chamber with an end-systolic volume). The ventricular inflow (atrioventricular region) and the outflow segment of the heart tube, on the other hand, retain a thick layer of cardiac jelly. These embryonic cardiac segments now act as functional heart valves because they prevent backflow of blood during the cardiac cycle [17]. They will become endocardial cushions which contribute to the definitive heart valves.

## 4. Heart Looping: From Tubular to Twisted Heart

Soon after the initial contractions, the heart tube bends and twists (loops) into a curved tube with its convex surface normally directed toward the right side of the embryo. When looping is complete, the heart has assumed the basic configuration necessary for further development into a four-chambered pump. There is a quite simultaneous onset of three different key changes: the first myofibrils appear [18], the first contractions occur [19] and looping begins [20], while effective blood flow does not start until a more advanced stage. At this stage the already complex evolving cardiac structure has a size of only about 300 microns.

### 4.1. Looping’s Phases

Looping consists of two main phases [21]: c-looping and s-looping. During normal c-looping (30–50 h in the chick embryo), the heart transforms from a straight tube into a c-shaped tube via two main deformation components: ventral bending and dextral (rightward) torsion. The original ventral surface of the straight heart tube becomes the outer curvature (convex surface) of the looped heart, while the original dorsal side becomes the inner curvature (concave surface). During c-looping, the myocardium thickens at the inner curvature and thins at the outer curvature [22]. During s-looping (52–68 h in the chick embryo), the atrium moves superior to the ventricle, creating the basic final form of the heart. Later (3.5–10 days in the chick embryo), septation divides the tube into four chambers. From an anatomical point of view we can identify four components of normal ventricular looping: (1) the ventral bending transforms a straight tube into a curved tube; (2) the torsion around its original cranio-caudal axis transforms a curved tube into a helically wound c-shaped loop; (3) the caudal shift of the ventricular segment that shortens of the distance between its cranial and caudal ends (s-looping); (4) the untwisting characterized by ventral and leftward shift of the proximal outflow tract [23].

Before looping, actin microfilaments in the outer myocardium cell layer are arranged in a net-like pattern at cell borders [24], while circumferentially oriented actin bundles and striated myofibrils begin to form in the inner myocardium cell layer. Looping begins when the first myofibrils appear and is hinged in dorsal mesocardium, driven by a differential hypertrophic growth in the developing myocardium that cause the heart tube to bend ventrally [25]. The bending component of c-looping is intrinsic to the heart tube, the torsional component of c-looping and the process of s-looping are influenced by external loads. C-looping is essentially complete before effective flow begins.

### 4.2. Hypothesis for Cardiac Twisting

External forces supplied by the splanchnopleure and omphalo-mesenteric veins appear to play prominent roles in the torsional component of c-looping. The omphalo-mesenteric vein may force the heart tube slightly rightward and then the splanchnopleure pushes it dorsally and farther rightward as the tube rotates about the dorsal mesocardium. Looping direction could be determined by a left-right difference in lateral forces exerted by the omphalo-mesenteric veins: the left omphalo-mesenteric vein is larger and exerts more pushing force than the right vein, causing the heart tube to twist rightward. Experiments have also shown that little torsion occurs when the splanchnopleure is removed. In 3D vision, the looped heart acquires a helical shape [25].

Cardiac looping has also another important meaning: it is the first expression of asymmetry in the embryo. At the very initial stage this asymmetry is due to nodal flow, that is the flow generated by ciliar elements in the node region, a transient midline structure formed during gastrulation. It has a ciliated pit with monocilia that have a peculiar microtubule arrangement and generate a clockwise, leftward motion of fluids [26]. This regulatory mechanism that the flow has in the extremely early stages of embryonic development remains also in the subsequent organogenesis.

## 5. Dynamics of Fluid Flow: Embryological Companion

The subsequent morphological development of an embryonic heart is dependent of a balanced interaction between a genetic program, fluid mechanical stimuli and the inter- and intracellular processes that link them. The dynamics of the flow at these early stages has been studied in zebrafish. The tube is narrow and only few blood cells can pass through it, necessarily positioning themselves in a linear manner (laminar flow). A blood cell takes 1050 msec to cover the entire tube in this pumping system [27]. The study of the movement of single blood cells suggests that at this stage the heart has a dynamic suction pumping mechanism [28]: the velocity over time of the blood cell depends by a suction, a relaxation and a compression phase of the heart.

### 5.1. The Transition from Continuous to Pulsatile Flow

At the same time as the looping process starts, there are profound modifications to the dynamics of the flow along the cardiac tube. The curvature of the system makes the passage of blood more difficult, but at the same time exploits the centrifugal force of the curvature itself [29]. To guarantee the efficiency of the flow, no longer guaranteed by the linearity of the system, it becomes essential to ensure the unidirectionality of the flow itself. Efficient pumping demands that retrograde flow be minimized. This requires some type of valvular system and endocardial cushions, which are the precursors of the heart valves, provide a clever way to satisfy all of the above design criteria during looping. Significant forward blood flow and pulsatile pumping become possible only when the endocardial cushions appear: as the contractile wave passes the cushions, they pinch off the lumen to prevent backflow.

During systole, the endocardial tube becomes compressed so that its lumen is almost completely occluded at end-systole. The diastolic expansions and systolic contractions of the ventricular myocardial wall of earlier heart tubes mainly run along the ventro-dorsal heart axis (c-loop), while during advanced stages of heart looping they run along the baso-apical heart axis (s-loop). The development of the ventricular segment is then characterized by the formation of trabeculated myocardium at its outer (apical) curvature [16].

Parallel to the unidirectionality of the flow, there is a need for an increased flow of blood: the developing heart increases its rate but mainly cavity volume [30], by further thinning cardiac jelly layer. Cardiac jelly recoils during diastole to help fill the heart. This more efficient system can now develop higher pressure. The measured flow rate increases from 0.02–0.15 mm^3^/s and the velocity of blood cells reaches 26 mm/s. It appears that the endocardial cushions act as primitive valves and induce a transition from peristaltic to pulsatile flow while increasing both pressure and flow rate.

### 5.2. The Mitral Valve: Embryological Teamwork

Among the valves of the heart, the mitral (between left atrium and left ventricle) has a particular function and is involved in many pathological states. In humans, the mitral valve develops between the 5th and the 15th week of embryonic life. The leaflet and chordal tissue derive from the endocardial cushion tissue lying on the inner surface of the AV junction. As the cushion tissue elongates and grows toward the ventricular cavity, it becomes progressively delaminated from the underlying myocardium, and the leaflet are transformed into a funnel-like structure completely attached to the myocardium. Then, perforations into the valve leaflet appear. The perforations grow and form the chordae tendineae, generated by the ventricular layer. The development of the papillary muscle takes place at the same time and is originated from the myocardium. As they increase in size, they progressively loose contact with the myocardial wall and stay in contact with the cushion tissue at their tip, finally connecting with chordae tendineae [31]. This development with different embryonic components marks the special interconnection between the mitral valve and the left ventricle that will guide their mutual functional behavior in the mature heart.

## 6. Permanent Link between Function and Form

The heart has to beat in order to support circulation well before its morphogenesis is complete. This is a fundamental passage that marks forever the indissoluble link between form and function of the heart, since both mature at the same time, and one could not develop without the other.

As stated, the peristaltic mechanics of the tubular heart is gradually replaced by a suction pump model. At the tubular stage, there is complete obstruction of the lumen by apposition of endocardium reinforced by cardiac jelly with 100% ejection fraction, which is never achieved at later stages. This geometry alternates between cylindrical, optimal for generating pressure, and spherical, optimal for volume displacement [32]. The conduction system at that stage has a well-defined pacemaker at the inflow, but the general speed of conduction is slow, correlating with the “peristaltic” mode of contraction.

Soon after the beginning of looping, morphological differentiation of myocardium along the cardiac tube becomes apparent. This includes disappearance of cardiac jelly in the atria and ventricles, coinciding with the process of development of trabeculae and pectinate muscles in the atria. The conduction system starts to show mature characteristics once the chambers are formed. The outflow valve function is exercised by a combination of close apposition of the outflow tract cushions and contraction of the slowly conducting myocardium of the outflow tract. Also the trabeculated embryonic heart observes the Frank–Starling relationship. As the conduction system develops, the transition from primitive base-to-apex activation of the ventricles to mature apex-to-base sequence becomes evident and correlates with ventricular septation. The fetal heart not only grows in size, but further develops spiraling myocardial architecture of the compact layer, with myofibrils of cardiomyocytes that tend to dispose along the maximal stress directions [33].

All these modifications take place while and because the heart has to cope with the growing metabolic needs of the new organism, without being able to wait for its own complete development.

## 7. Mature Heart: A Summary to Read and a Lesson to Follow

The left ventricle is a remarkable system characterized by the unique ability to translate 15% linear sarcomere shortening into ejection fractions of greater than 50% and wall thickening greater than 30%. This is obtained by a multidimensional network of myocardial fibers with different angles in the various layers of the myocardium, if observed in cross sections. Predominant orientation of myocardial fibers varies with wall thickness, and the pattern is reminiscent of multiple concentric layers of hoops [34]. The histologic cross-section probably is not the best method to describe the structure of the myocardium. The seminal works by Torrent-Guasp [35] and Buckberg [36,37,38] demonstrate that a 3D view of this complex fibers’ system is the best way to describe the “myocardial continuum” defined by Sponitz [34], although a true depiction of the fibers’ arrangement is very complex. Myocardial syncytium starts from myocells placed side by side in a staggered way and then organized into groups of fibers which in turn form larger bundles. Everything is complicated by the orientation of the fibers that changes within this histological mesh, with a variable angle within the myocardial thickness from the epicardium to the endocardium. Some high-level imaging techniques [39] can now help to visualize this unique muscular arrangement.

The shortening of the fibers that intersect each other with different angles of direction works like a cooperating system that gets a contraction, as Harvey said, in all directions and a shortening along the major axis. The interlaced structure allows ventricular systole with low energy expenditure, thanks above all to a fundamental parameter such as the torsion of the left ventricle. The oblique fibers rotate the ventricle on its axis in the opposite direction between the apex and the base. This “squeezes” the ventricle like a “wet cloth”.

As mentioned, the growth of the heart occurs simultaneously with the development of its pump function. This feature of having to ensure the circulation of the evolving organism while it has not yet finished its own organogenesis is the determining fact of what we see today: the structure and the function are strictly related since they developed simultaneously. In this setting, the growing needs of circulating blood of the embryo condition the incremental development of the myofibrils and myocardial fibers, the maturation of a more suitable and capillary electrical conduction system, its own coronary support circulation, a suitable geometry of ventricular chambers etc. Embryologically speaking, what occurs is in practice a sort of “on demand” development, according to the needs that the embryo “asks” to the organ that assures its perfusion.

**Figure 2:**
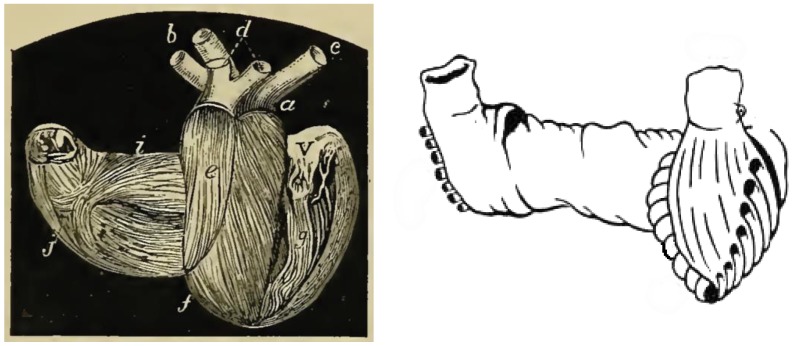
J. Bell. Pettigrew, The physiology of circulation, Publisher Macmillan, London, 1874, Figure 139, page 215, engraved on wood, (left, public domain electronic copy at https://archive.org/details/b20401619). Note the clear similarity with the Torrent–Guasp myocardial band, 1998 (right, adapted from http:// www.torrent-guasp.com/pages/vmb%20form.htm).

Early scientists framed the complex structure of the heart since the time of Erysistratus (304–250 BC), a Greek anatomist and royal physician who described the valves of the heart, and concluded that the heart was not the center of sensations, but instead it functioned as a pump. Later we can find a precise description of the course of the fibers and its usefulness in the work of William Harvey [40], Lower [41], Vesalio [42], Pettigrew [43] (Figure 2), Lancisi [44], Mall [45], until the theory of the muscular band of TorrentGuasp.

If one envisions the looping process of the primitive heart tube while it contracts, it wraps itself, thickens its walls, divides into four different pressure chambers and pumps blood into an increasingly complex organism, it is conceivable how its final form is the suitable result of this process. The heart keeps on contracting as it does from its twenty-first day of life but with a structure and shape that continuously changes over time. The perinatal period is that which definitively shapes the myocardial mass as an adaptive consequence of the major haemodynamic changes occurring after birth that generate major parietal stress variations and parietal remodeling in different parts of the heart. The plasticity of myocardial tissue arrangement still present in this phase allows such adaptation. The ventricular myocardial architecture becomes more inhomogeneous, revealed by a variation of the regional isotropy index [46].

## 8. What the Heart Remembers of Its Development

The knowledge of cardiac function has progressed in a very fragmented and poorly integrated way among the different involved disciplines, at least until the high level of knowledge currently achieved. The need for advanced technologies has certainly slowed down this process. However, now the knowledge reached in embryology, physiology, pathology, in medical and surgical treatment and in specific instrumental examinations allows a more complete and integrated vision of cardiac function. From the whole sum of knowledge we can deduce that cardiac morphology and its related function maintain the fundamental characteristics of its development. We then list major characteristics of the mature heart that derive from its crucial embryonic steps: laminar flow, sequential electrical activation of contraction, elliptical geometry of ventricular chambers, fibers disposition, left ventricular torsion and valve function with special mention for the mitral valve. We add a clinical perspective for each of them that summarizes the state of the art of relative pathological states and outlines possible future improvements. Some of these perspectives are purely speculative but for most of them there are already important early evidences.

### 8.1. Laminar Flow

**Figure 3:**
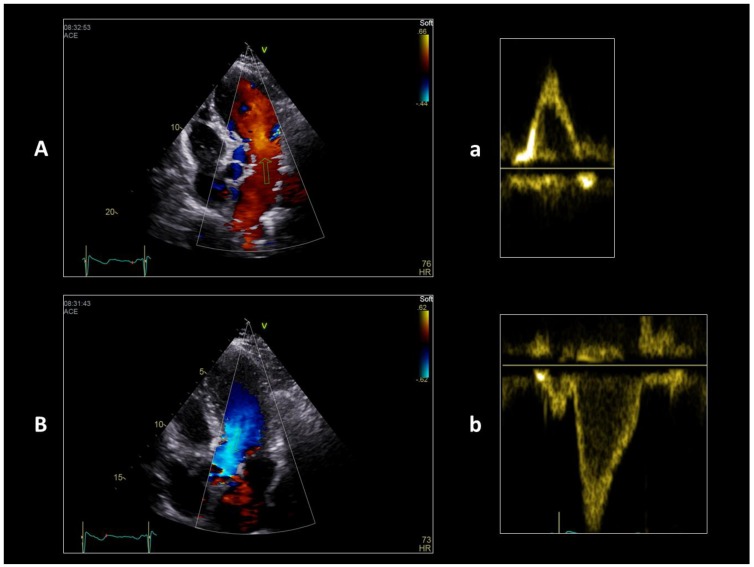
Echo color Doppler (**A**,**B**) and Pulsed Doppler (**a**,**b**) study of diastolic (**A**,**a**) and systolic (**B**,**b**) intra-ventricular flows in a healthy volunteer. The color homogeneity and the black areas under the pulsed curves indicate a laminar flow.

If you imagine bending a straight tube and keeping a linear flow within it, the final shape must have as little bending as possible. The final conical shape of the left ventricle (and also of the right one) recalls a straight tube with a single fold at the apex. The inflow and outflow sections are connected by an acute angle at the apex of the heart. This configuration allows maintaining a laminar flow in both anatomical areas, obtains the almost complete occlusion of the ventricular chamber and exploits the apex as a sort of parabolic curve to favor the blood exit in systole. In this way, also in the elliptical shape of the mature left ventricle, in normal conditions, the blood flow maintains a linear pattern, just following the curved apical region. Although it is an important variation in the linear aspect of the blood path, the elliptical shape of the ventricle with the presence of the cardiac apex has been positively selected exploiting the diastolic vortex, an important parameter in the dynamics of the blood. The profound modifications due to the looping also induce a chiral [47] asymmetry of the flow in the cavities which facilitates the separation of the inflow from the outflow [48]: the left ventricle works in practice according to a first-in first-out mode and in physiological conditions the complete passage of a certain amount of blood ends within about nine beats. The first-in first-out mode is still the mode of expulsion of blood from the linear heart tube. As is visible in echo Doppler study of the flow inside the ventricle (Figure 3, Video S1), it has a double linear direction in the cardiac cycle, down from the mitral valve to the apex in diastole and up from the apex towards the aortic valve in systole.

**Figure 4:**
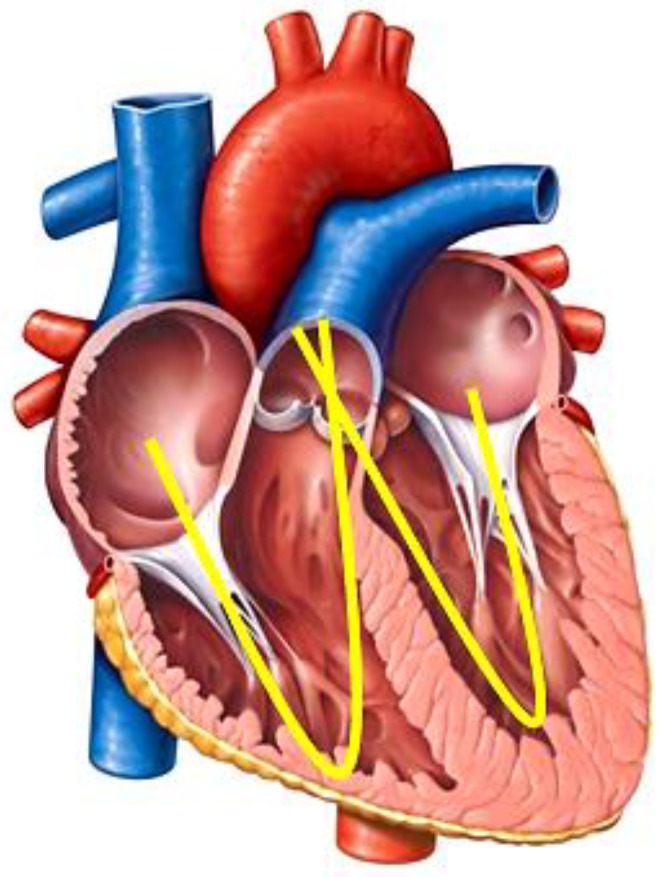
The “W” flows inside the heart (yellow lines).

The two components of the flow are both linear and are connected by the apical vortex during the diastolic phase, which accounts for the transportation of 10% of the whole filling volume. The septation of the ventricular cavity doubles this configuration in a “W” shape of the left and right flows of blood (Figure 4).

Apart from the different flow vortices that are generated inside the heart due to the different morphology of the cardiac chambers, this behavior could be considered a remnant of the original linear flow in the cardiac straight tube. These two main flow components are also synchronous with the activation of the descending and the ascending muscular bands. The advantages of linear flow are well known and are positively selected during the growth process of the heart [49]. Thanks to this normal configuration the heart barely needs approximately seven Watt to perform its function (less than half the power consumption of an implantable biventricular assist device) [50].

#### – Clinical Impact

In pathological situations (valve disease, cardiomyopathy etc.), the linear flow is disturbed with negative consequences on the ventricular efficiency (chamber dilation, higher viscosity, higher blood stress) [51]. When flow becomes turbulent, the fluid elements do not remain confined to definite laminae, but rapid, radial mixing occurs. A much greater pressure is required to force a given flow of fluid through the same conduit when the flow is turbulent than when it is laminar, so the heart must do considerably more work to generate a given flow if turbulence develops [52,53]. Valve diseases mainly alter ventricular flow, but also changes in ventricular shape (and volume) can affect the turbulence of the flow, simply by enlarging the section of the conduit where the blood passes or altering the diastolic vortex. In dilated ventricles there is a higher loss of kinetic energy during diastole than in healthy subjects [54] and a higher blood residence time. When a pathological condition is repaired, e.g., with valve replacement, the flow can return to normalcy only if the ventricular cavity has not changed due to pathological hemodynamics. If the pathological condition has lasted for a long time, the ventricular (or atrial) changes involved (hypertrophy, dilation, deformation) remain without regressing. What is important to note here is that the guidelines relating to the surgical treatment of cardiac pathologies report criteria of indication to the intervention that often coincide (or even exceed) with points of no-return of the alterations occurred. To obtain the best result, it would be necessary to intervene when the physiological range of hemodynamic modifications is still present. Therefore the study of flows into the ventricle would be another parameter to be included in the evaluation of the severity of a disease. Among new techniques, echocardiographic vector flow mapping [55] can calculate the dissipative energy loss derived from the velocity vector field of intra-ventricular blood flow, is considered to reflect the efficiency of blood flow and could be an indicator of left ventricular function.

### 8.2. Sequential Electrical Activation

**Figure 5:**
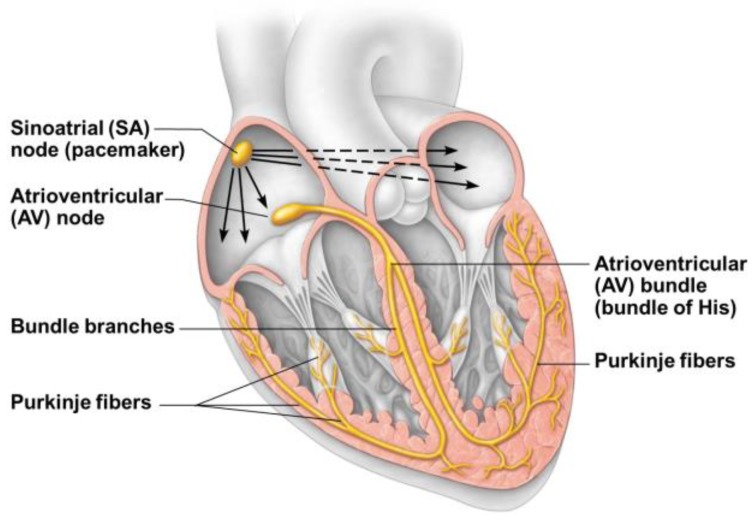
Adult heart conduction system that depicts the original single starting point in the ex sinus venosus area (sino-atrial node), the atrioventricular node and the subsequent more capillary branches that follow the myofibrillar development of cardiac ventricles.

The conduction system maintains its embryonic characteristics of an inflow kickstart center (sinus node) and a conduction pathway that sequentially (“peristaltically”) activates all the myocardium following the muscular bands (looped myocardium) development and evolution. Given the looped and twisted structure of the latter, the peripheral branches of the conduction system have a reversed direction towards the base of the heart (Purkinje fibers), following the bended and twisted ventricular portion of the old cardiac tube (Figure 5).

#### – Clinical Impact

The importance of resynchronization therapy is well known in heart failure treatment as well as in late definitive pacemaker de-synchronization. Sometimes the only resynchronization is able to reduce a mitral regurgitation on the basis of a better wall motion (papillary muscles synchronism). De-synchronization alone is sufficient to cause irreversible heart failure. Nevertheless, at the best of its results resynchronization therapy has still a 30% of non-responders [56]. That could reasonably be due to the severe challenge (until now) to choose the exact place where apply the electrical synchronized stimulus along the myocardium, in order to restore the normal pathways of sequential conduction and myocardial activation. Some recent studies [57,58] and a trial (LV Endocardial CRT for Patients With Intermediate QRS Width (EndoCRT), no. NCT03573427) focus on this issue adding one or more leads to improve synchronism or choosing the endocardium as a pacing site. This research demonstrates the importance of replenishing the Purkinje terminal network (multiple stimulation sites instead of the single one used so far in a postero-lateral cardiac vein) but will also require an anatomical-functional study to make sure that the stimulus arrives in the correct point of the myocardial bands according to the various settings of dyssynchrony. Probably, the application of the electrodes should also be surgical and not only endocavitary once the correct resynchronization site/sites has been identified.

### 8.3. Elliptical Shape (Small End-Systolic Volume)

An elliptic cross-section has a higher mechanical efficiency compared with tubular cross-section, and the elliptical shape of the final heart assures the highest efficiency for blood expulsion with a narrow cavity squeezed nearly completely. Elliptical shape guarantees a laminar flow, offering the smallest diameter in the folded mature heart. Need for feeding a wider organism, higher pressure mature system and the creation of a ventricular chamber made necessary the development of and end-systolic volume, lowering the 100% ejection fraction of the embryonic heart. That is the reason why end-systolic volume must be the smallest possible and why it was soon recognized as a sensitive marker of left ventricular dysfunction [59], a negative prognostic factor after a myocardial infarction [60] and an indicator of poor result after ventricular reconstructive surgery [61].

#### – Clinical Impact

The results of STICH trial [62], even if reported without reaching the volume reduction endpoint required by the study protocol, confirm the limits of surgical left ventricular reconstruction as performed until now, based on mere volume reduction [63]. This surgical procedure should be reengineered, considering all the physiological parameters and characteristics of normal left ventricular function, adding in particular the study of fiber re-orientation and the absolute care of the critical role that physiologic ventricular shape plays. STICH study’s subgroups, in which the volume reduction obtained the required amount, demonstrate a better outcome if end-diastolic and end-systolic volumes were less than 90 mL/m^2^ and 50 mL/m^2^, respectively [64]. In our recent clinical series (see next paragraph), we obtained a stability of LVESV during time starting from a surgically reduced cavity similar to normalcy (mean value 38 ± 14 mL/m^2^), with an increase during 7-year follow-up of only 8–10 mL/m^2^. The elliptical shape is fundamental in obtaining a small ventricular volume: in a box-shaped or spherical heart would be impossible to maintain volumes at a low value and have at the same time the squeezing systolic efficiency [65].

### 8.4. A Very Special Twist

Left ventricular torsion is essential for efficient ejection (Video S2), and many studies confirm its importance as a sensitive marker of ventricular function in physiologic and pathologic settings. Torsion appears to be a critical link between systole and diastole, with elastic energy stored during systole and then released with untwisting during isovolumetric relaxation. The twisting motion of the left ventricle about its long axis results from the contraction of the opposite, obliquely oriented epicardial and endocardial fibers that generate two opposite rotations: the basal one, clockwise and the apical one, counterclockwise [45]. From the Torrent-Guasp theory of the sequential activation of descending and ascending myocardial bands, the twisting and untwisting of the left ventricle assumed an even more important and active role in cardiac mechanics. Mainly the diastolic function, the most hidden and less defined part of cardiac cycle, could be more clearly understood if partially linked to an active contraction of the ascending muscular band [38]. Torsion movement derives from the more peculiar passage of cardiac embryogenesis and guarantees a high contractile efficiency, since the interweaving of the fibers acts by multiplying the contractile effect with low energy expenditure [66,67].

#### – Clinical Impact

Torsion is altered in all cardiac pathologic states, with different degrees according to the severity of disease and its alteration is reversible if the pathologic injury can be removed [68,69]. Ischemic cardiomyopathy is a more irreversible disease, in which the myocardial tissue structure is completely destroyed in the infarct region. When the infarcts tissue is wide enough to alter 3D myocardial structure mainly in the apical region, rotation of the apex and global torsion are reduced or nullified as a consequence [70]. The reversibility of this lesion is difficult to foresee, due to the irreversible loss of myocardial tissue. The structural modification following a myocardial infarction, and the consequent loss of myocardial tissue and continuum, makes the renewal of torsion quite unexpected. In facts, the restoration of ventricular torsion, though postulated, was never demonstrated before with any technique of ventricular reconstruction [71,72,73,74].

We personally hypothesized [75] and then devised [76] a new surgical ventricular restoration technique (KISS procedure) with the aim to restore residual fibers disposition, on the evidence that normal fibers disposition remains intact in the perinecrotic areas [77]. Thanks to a special suturing technique that brings together the still normal fiber bundles in a physiological arrangement, we were able to obtain the renewal of torsion also in case of severe, end-stage ischemic left ventricular dysfunction [78,79] (Video S3).

**Figure 6:**
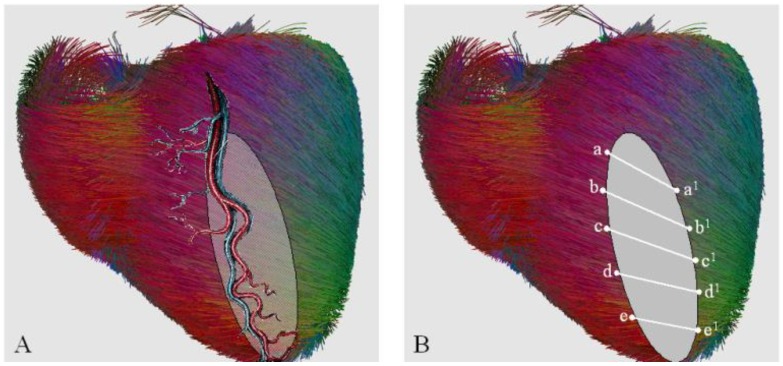
Coronary vessels and fiber orientation. (**A**) Epicardial course of coronary arteries crossing fiber orientation. The shaded area depicts transmural necrosis induced by coronary vessel occlusion which concerns a sector of myocardium made by several strata of differently oriented myocardial fibers. (**B**) At the border zone, still normal myocardium with still normal fiber orientation interlaces with the necrotic tissue. Necrotic tissue discontinues contiguity of myocardial fibers, although they remain normally oriented in the thickness of normal myocardium. Once fibrotic tissue is removed, the surgical suture matches the correspondent points (“a” with “a1”, “b” with “b1” etc.) restoring the contiguity of residual, normal bundles of fibers. Adapted from the reference [76]. Fiber picture was obtained by the DTI Track module of MedINRIA open source software (download at https://med.inria.fr/).

This surgical reconstruction was based on current human diffusion tensor imaging of myocardial fiber orientation (Figure 6). This knowledge was integrated with wider studies on mammalians’ heart with the aim to figure a statistical atlas of cardiac muscle fiber architecture [80]. Recently, some clinical applications of diffusion tensor imaging have been reported in literature [81,82,83,84], confirming the interest in this complex but high-resolution technique.

**Figure 7:**
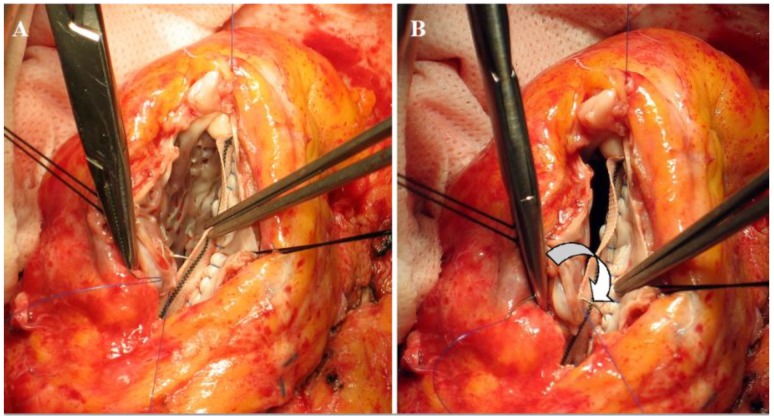
Surgical technique to re-approach fibers’ bundles in a physiologic disposition. Panels (**A**) and (**B**) depict the stretching of the suture that approaches and fits the lateral ventricular wall to the patch. The arrow in panel (**B**) shows how the suture redirects fiber orientation, approaching a farer point of the myocardial wall to a nearer point on the patch. Adapted from the reference [76].

With our surgical suture we surely are not able to restore a microscopic, histological contiguity of myocardial layers, but the renewal of torsion shows that the final effect of rotation can be obtained even at a gross, tissutal level, if the remaining still normal tissue is re-hinged to a near-physiologic disposition (Figure 7).

The recovery of the torsion is so specific that it can only be due to the matching of the structurally normal residual fibers, excluding the non-contractile fibrotic zone. We observed the recovery of apical rotation just after the surgical correction in the operating room and then confirmed it with a comparative echocardiographic 2D speckle tracking study. The early timing of torsion restoration testifies a mechanic correction due to surgical treatment and not to a positive remodeling occurring over time.

Rapidly evolving imaging techniques will make possible a preoperative exploration of cardiac fiber mapping, giving the surgeon the possibility to plan a more precise surgical operation focused on the restoration of normal fiber orientation. Some of the negative results obtained so far could be due to the involuntary incorrect reconstruction of the residual bundles of fibers.

The renewal of torsion reinstates to the ventricle part of that energy efficiency lost through dilatation and dysfunction, and this can translate into a significant clinical improvement, especially in ventricles that work at a critical level in the Frank–Starling relationship.

### 8.5. Valved Tube, Valved Heart

One of the fundamental steps for cardiac development is the guarantee of a one-way flow obtained through the formation of the valves. Their efficiency remains essential for the proper functioning of the heart. Valve diseases are known to significantly reduce cardiac function over time, so that if the correction arrives too late in the natural history of the disease it is no longer possible to recover normal contractility and efficiency even after valve repair or replacement. It is also known that the negative consequences on the heart differ according to the diseased valve and the type of disease: valve regurgitation is worse tolerated than valvular stenosis and the mitral valve disease is more complex than that of the aortic valve. This is also an embryological memory: the regurgitation nullifies the unidirectionality of the flow, which is precisely the reason why the development of the valves has been positively selected. Valvular regurgitation not only wastes contractile energy, but mainly wastes part of the antegrade flow, so cardiac mechanics suffers from a double loss of efficiency, energetic and functional. In valvular stenosis, instead, the myocardium must spend more contractile energy to overcome the valve obstacle but, at least for a long time, this higher energy expenditure ensures an adequate systemic flow.

#### – Clinical Impact

The guidelines for the treatment of valve disease are still very indulgent on the precocity of the treatment itself [85]. In fact, the treatment is indicated only when the pathology is very advanced, and never in its early stages, based on general parameters of cardiac function (ejection fraction, transvalvular gradient etc.) without a complete and thorough view of all that is altered in a heart with valvular disease (intraventricular flow, interstitial fibrosis, fibers’ arrangement, volumes, etc.). The lack of optimal prosthetic substitutes and the procedural risk certainly favor this more cautious attitude. However it should not be forgotten that the good outcome of the correction of the valvulopathy depends on the residual myocardial efficiency and on the still present possibility to recover what was lost during the pathological state. A more precise identification of the negative consequences of a valvular disease on the heart muscle is desirable: the energy consumption, the analysis of flow dynamics, the amount of intramyocardial fibrosis [86,87], are all parameters that should help in defining the degree of valvular disease and the reversibility of the resulting myocardial pathology. It should be remembered that the changes implemented by the heart to counteract the negative effects of a valve disease reduce the cardiac efficiency anyway. For example, many echocardiographic medical reports of aortic stenosis quote the phrase “presence of adequate parietal hypertrophy”: parietal hypertrophy can be defined as “necessary” to overcome the transvalvular gradient but it nonetheless denotes a pathological state of the myocardium with disarrangement of the 3D structure of the fibers, higher energy consumption, greater flow turbulence, etc. Therefore its “adequacy” often coincides with irreversible modifications.

### 8.6. A Very Special Valve 

There is a big difference in the embryological development between atrioventricular (inflow) valves (mitral and tricuspid) and outflow valves (aortic and pulmonary). The former in fact grow and implant inside the ventricle and are connected to it, i.e., to the moving muscle. The outflow valves instead are implanted in the tributary vessel and are thus disconnected from the systo-diastolic movement of the myocardium. This makes the mitral valve (and tricuspid) very special: its proper functioning does not depend only on the integrity of its leaflets but also on the conditions of the left ventricular muscle. Abnormalities of contractility, perfusion, synchronism, fibers’ disposition and ventricular geometry can all interfere with its normal functioning [88].

#### – Clinical Impact

The complexity of the mitral pathology finds its maximum expression in left ventricular dysfunction and heart failure, both idiopathic and ischemic. Especially in ischemic heart disease, thousands of articles have been written about the best treatment to follow. The long diatribe if repair or replacement was preferable ran ashore in some recent key studies that demonstrated the poor results of the repair of ischemic mitral disease [89,90,91]. This evidence, although predictable bearing in mind the mitral-myocardial interaction, has amazed the cardiac surgery world but has also allowed to correct the guidelines on the subject. Today the mitral replacement in case of ischemic cardiomyopathy with a surgical technique that maintains the chordae tendineae system, has a recommendation of type IIa, B-R [92]. Chordal sparing technique was introduced by Lillehei in 1964 [93] and plan the replacement of the mitral valve preserving all the chordal apparatus that is sutured together with the prosthetic ring, maintaining the mitral-myocardial connection and assuring the valve competence. Although the valve is replaced, this solution allows to split the valve’s competence from the interaction with the dysfunctional ventricular wall and to maintain the facilitating effect on the contraction guaranteed by the integrity of the chordal apparatus.

Another theory of mitral repair has been unconfirmed over time due to poor results [94,95,96], namely the restrictive anuloplasty [97,98], i.e., the application of a prosthetic ring approximately 4 mm narrower than the real size of the mitral ring, in the idea that this could reduce one of the ventricular diameters (the basal diameter). The failure of this technique is linked to the fact that it acts only on one of the structural and functional parameters that determine secondary mitral regurgitation (a common limit to mitral repair on the whole), while we have seen that the correct functioning of the mitral valve is linked to a complex set of anatomical and functional parameters. The mitral valve “remembers” its complex embryological origin deriving from four tissue components and its normal function remains connected to the entire structure of the left ventricle, not only to its moving elements.

## 9. A Multidimensional Organ

The heart is undoubtedly the beating engine of our life. It can stand the heaviest proofs, bare the most difficult situations and face the worst diseases. Unfortunately, the heart loses its morphogenic potential at the end of its development. In the adult heart, new mitoses, though present, are not enough to sustain a regenerative capacity of the organ. The pluripotential cells that contribute to the embryological development of the heart are definitively differentiated at its end and this process becomes irreversible. This means that the adult heart will work in the set-up present at the end of its embryological development and that the pathological changes will have to be counteracted using only the final structure of the heart. Therefore the heart can only keep the memory of its development and not retrace its various passages. This makes the organ we see an excellent final product to be stored jealously and to be known at our best in order to be able to treat its various pathological states. The complex structure of myocardial tissue is at the base of its physiology, and we must keep this in mind in everyday clinical practice.

The heart is truly a multidimensional organ.

Its physiological function is based on several different parameters and characteristics: histology, tissutal disposition, endoplasmatic reticulum, myocardial coronary and sinusoidal circulation, conduction system and electrical sequential activation, intra-ventricular synchronization, chamber geometry and volume, fiber orientation, opposite rotation of apex and base generating torsion, elliptical shape, systo-diastolic alternation, filling pressure and volumes, afterload pressure, electrolytes exchange, metabolic needs etc. Each one of these parameters is a tile of the complex puzzle of heart function. Different heart diseases can alter one or more of these parameters, developing several functional and clinical pictures, whose therapy must take into account all the parameters involved. If we try to address singularly each of the normal parameter, we cannot be able to succeed in the treatment of the disease because all parameters are tightly interlaced. When a disease damages the heart, it damages a set of interconnected anatomical structures that have knock-on effects on various functional parameters: a multidimensional organ needs a multidimensional, contemporary treatment.

Many examples support this close, multidimensional interconnection.

Even only the change in heart rate modifies the pattern and the timing of endoventricular flow vortex, increasing or decreasing its kinetic energy and its position relative to the mitral valve [49].

The two opposite movements of apical and basal regions generating torsion have a turnaround point of twist to untwist motion pattern located coincidentally at the papillary muscle level, with three rotation patterns: counterclockwise rotation, clockwise rotation, and counterclockwise to clockwise rotation. The exact level at which this happens in each heart is important for the interaction with mitral valve function [99].

Preservation of the LV systolic torsional profile may be a helpful predictor for post-mitral surgery survival and reverse remodeling in patients with non-ischemic secondary mitral regurgitation with narrow QRS, and patients who received resynchronization prior to mitral surgery had poor post-surgery survival, whereas those who received resynchronization at the time of mitral surgery had better survival [100,101].

A new design of artificial heart has been proposed which mimics global left ventricular wall deformation patterns in terms of circumferential and longitudinal contraction, as well as torsion: it will likely result in more physiological intracavitary blood flow patterns and higher efficiency [102].

## 10. From “CARDIOmyopathy” to “FIBERmyopathy”

A new concept, a new vision of the heart can arise from the knowledge we have today: we should no longer be thinking in terms of *cardiomyopathy* as a disease of the heart muscle as a whole, but in terms of *fibermyopathy* with reference to its intrinsic structure. In this perspective, perhaps many of the clinical results reported in literature will have to be reinterpreted, in a more comprehensive and correct view of what we treat interventionally, surgically and pharmacologically every day.

How many studies on mitral repair take into account the turnaround point of twist to untwist motion pattern? How many pharmacological studies care of fibers’ disposition to judge the medical effect? How large are end-systolic volumes in heart failure studies that select patients with 30% ejection fraction [103]? The more we deepen our knowledge of a field, the more we should deepen its study criteria to obtain appropriate results. The acquired knowledge must guide an in-depth scientific vision in future papers and guidelines on the topic and a complete, farsighted therapeutic conduct able to ensure the physiological correction of cardiac pathologies. 

I entrust the conclusion to the words of the final remarks of Pettigrew’s Physiology of Circulation Lecture: “The scheme of the course and direction of the fibers as summed up, while it greatly facilitates the comprehension of the general principle involved in the ultimate structure of the ventricles, harmonizes in the most perfect manner with all that is at present known of the heart’s movements—those movements apparently so simple, and yet so difficult of analysis. How far I have been right in doing so, time and advanced Physiology will determine”.

As scientists, we should keep on studying and applying the complex structure of the heart remembering each of its constituent elements, to better understand, appreciate and care of this *instrumento mirabile* that each of us owns.

## Supplementary Materials

The following are available online, Video S1: Echocardiographic flow pattern of the left ventricle in a healthy volunteer, slow motion. The diastolic (red) and systolic (blue) components of the flow are both laminar, with no turbulence (see the homogeneous full color, no mosaicism, only normal aliasing for systolic flow acceleration). Video S2: Surgical view of a normally contracting heart. The torsion motion is evident in the apical region, which is raised up to a better view (slow motion). Video S3: Pre- and post-operative echocardiographic standard and 2D speckle tracking (ST) study in a 73 year-old female patient operated by KISS procedure. Standard echo shows key modifications obtained by the described technique: elliptical shape, small volume, new apex and the reduction in tenting of the mitral valve due to the restoration of a near-normal ventricular chamber. 2D ST echo shows the preoperative abnormal clockwise rotation of the dilated apex with fibers disarranged and the postoperative near-normal counterclockwise rotation of the restored new apex with fibers re-oriented. The two controls differ by a few days.
